# Effects of Dietary Red Grape Extract on the Quality Traits in Juvenile European Sea Bass (*Dicentrarchus labrax* L.)

**DOI:** 10.3390/ani13020254

**Published:** 2023-01-11

**Authors:** Simona Tarricone, Nicolaia Iaffaldano, Maria Antonietta Colonna, Francesco Giannico, Maria Selvaggi, Anna Caputi Jambrenghi, Michela Cariglia, Marco Ragni

**Affiliations:** 1 Department of Soil, Plant and Food Sciences, University of Bari Aldo Moro, 70125 Bari, Italy; 2 Department of Agricultural, Environmental and Food Science, University of Molise, 86100 Campobasso, Italy; 3 Department of Veterinary Medicine, University of Bari Aldo Moro, 70010 Bari, Italy; 4 Gargano Pesca Società Agricola Consortile Arl-Società Benefit, Porto Alti Fondali, 71043 Manfredonia, Italy

**Keywords:** Sea bass, polyphenol extract, red grape, grape seed extract, fatty acid profile

## Abstract

**Simple Summary:**

The need to satisfy the growing demand of aquaculture production has led to an increase of intensive fish farming techniques that are held responsible for the occurrence of various stressors that affect the health of cultured animals. Polyphenols are plant-derived compounds with known biological activities and positive influences on the performances and immunity of fish. The aim of the present study was to evaluate the effects of dietary inclusion of a polyphenol extract obtained from Nero di Troia red grape, tested at two concentrations, on the quality of juvenile farmed sea bass (*Dicentrarchus labrax*) fillets. The two diets containing polyphenol extracts overall improved the fish quality features; in particular, dietary inclusion of grape extracts lowered the concentration of total fat and saturated fatty acids along with the Atherogenic index, with benefits for human health. No differences arose between the two GPE concentrations tested; therefore, the polyphenol-rich extract showed its antioxidant effectiveness in preventing lipid oxidation and enhancing fish fillet quality already at the lower concentration. Further investigation is needed in order to study the effects of the experimental diets on fish performances, immune status, and flesh quality upon achievement of the sea bass market size.

**Abstract:**

Intensive fish farming is responsible for the occurrence of various stressors that negatively affect the health of cultured animals. Polyphenols are plant-derived compounds with biological activities and positive influences on the performances and immunity of fish. The aim of the present study was to evaluate the effects of dietary inclusion of a polyphenol extract obtained from Nero di Troia red grape on the quality of farmed sea bass (*Dicentrarchus labrax*) fillets. Three diets were tested: control (*n* = 90) received a conventional feed, whereas the two experimental groups (*n* = 90 each) received the control feed supplemented with the red grape polyphenol extract (GPE) at the concentration of 100 (GPE 100) or 200 mg/kg (GPE 200). The two GPE diets lowered (*p* < 0.05) the red (a *) and yellow (b *) indexes, fillet hardness, and total lipid content. Chewiness, concentration of saturated fatty acids, and the Atherogenic Index were higher (*p* < 0.05) in the control group; GPE diets increased (*p* < 0.05) the polyunsaturated fatty acids content. Furthermore, the concentration of malondialdehyde was lower (*p* < 0.05) in fillets of the GPE groups, thus confirming the antioxidant effect of the red grape extract and its effectiveness in preventing lipid oxidation.

## 1. Introduction

Global aquaculture production quadrupled between 1990 and 2017, and nowadays farmed fish accounts for one-sixth of the worldwide consumption of animal protein in the human diet [1]. The need to satisfy the increasing demand of aquaculture production has led to an increase of intensive fish farming techniques in order to improve growth rates [2]. Intensive aquaculture systems, however, are responsible for the occurrence of various stressors that negatively affect the health of cultured animals and predispose them to diseases and increased mortality, resulting in economic losses [3]. As a consequence, several drugs and chemicals, such as antibiotics, have been used over time in fish farming. The presence of the residues of these substances in fish represents a major concern for human health [4], thus leading the European government agencies to regulate the reduction of drugs and chemicals in aquaculture [5]. Therefore, research attempts alternative to the use of drugs and chemicals, including dietary treatments, have been made in order to find strategies able to improve the immune status and health in farmed fish [3]. Different nutritive and non-nutritive compounds/additives among which amino acids, essential fatty acids, phospholipids, vitamins, minerals, carotenoids, synthetic chemicals, and biological derivatives of different origin have proven to exert stress-mitigating effects in aquaculture fish species [4]. Hence, the dietary administration of some plant-derived extracts, rich in bioactive molecules, is a recent strategy attracting growing interest [6,7,8,9,10], since these compounds show antioxidant, anti-inflammatory, immune-stimulating, anti-microbic, and microbiota regulating effects [11,12].

Polyphenols have well-known biological activities and potential health benefits [13,14]. Over the past decade, many studies have investigated the health effects of polyphenol-rich red wine and purple grape juice in both human and animal models [15,16,17]. Polyphenols have oxygen- and nitrogen-derived free radical scavenging properties, modulating antioxidant enzymes and cellular redox transcription factors [18]. In particular, the protective effects of polyphenols consist in the continuous removal of various reactive oxygen species (ROS) from cells, such as singlet oxygen, peroxynitrite, and hydrogen peroxide, in order to maintain healthy metabolic functions [19]. 

Several types of polyphenolic compounds such as flavonoids, phenolic acids, and lignans, are present in fruits and vegetables and in plant-derived beverages, such as tea, purple grape juice, and red wine [20,21,22]. As for grapes, the polyphenol concentration widely varies within grape varieties, being also influenced by viticultural and environmental factors along with the wine-making process [21].

The skins and seeds of grapes are sources rich in polyphenolic compounds, including flavonoids and non-flavonoids [23,24]. About 60% of the total polyphenols are present in the grape seeds, while more than 70% of grape polyphenols are not extracted during the wine making process and remain in the pomace. Among flavonoids, anthocyanins are one of the most potent antioxidants and are primarily located in the berry skin, mainly in the outer layers of the hypodermal tissue, to which they confer colour, having a hue that varies from red to blue. Although the effects of wine polyphenols have been intensively studied, it has been demonstrated that the synergistic effect of phenolics with other wine components such as ethanol and organic acids, along with low pH, may be a source of variability of wine bioactivity [25,26,27]. Moreover, the antimicrobial activity of specific components of wine is still a matter of debate. According to some authors, in both animal models and humans, polyphenols from red grapes are endowed with better antioxidant and anti-inflammatory activities, being able to act on the equilibrium of the immune system [28,29,30]. While the effect of phenolic compounds from red and white wines has been associated with a beneficial impact on the performances and immunity of fish [20], only few investigations on the antioxidant and anti-inflammatory effects of grape polyphenols in farmed fish have been conducted [31,32].

A study carried out within a previous research project reported that the dietary inclusion of a red grape polyphenol-rich extract in sea bass determined lower levels of intestinal proinflammatory cytokines along with larger amounts of splenic interferon (IFN-𝛾), thus showing the occurrence of a strong and protective adaptive immune response that contributes to a greater maintenance of fish health [30,31].

Hence, the aim of the present study was to evaluate whether the dietary inclusion of the same grape polyphenol-rich extract would affect some quality traits of juvenile farmed sea bass (*Dicentrarchus labrax*) fillets.

## 2. Materials and Methods

### 2.1. Preparation of Feed and Dietary Regimen

The fish diet consisted in a conventional feed whose ingredients and chemical and fatty acid composition are shown in Table 1. The experiment was carried out in triplicate: the control group (*n* = 90) received the conventional feed, whereas the two experimental groups (*n* = 90 each) received the control feed mixed with two different concentrations of a red grape polyphenol extract (GPE). Canosina red grape from Nero di Troia is an autochthonous *Vitis vinifera* (L., 1753) grape cultivar that grows in Apulia (southern Italy). It is characterized by thick-skinned and small-sized berries. Frozen seeds from berries were extracted by percolation using ethanol/water (70:30). Then, the extract was first analysed by means of liquid chromatography with diode array detection to define the polyphenol composition. Thereafter, the extract was purified on a synthetic adsorbent brominated resin, and the percentage of polyphenol content was determined, as described by Magrone and Jirillo [30]. As far as the polyphenol content of the red grape extracts is concerned, percentages were the following: proanthocyanidins (101.8%) and catechins plus epicatechin (10.37%). In this experiment, two concentrations of the GPE were used: 100 (GPE 100) or 200 mg/kg (GPE 200), respectively.

### 2.2. Fish Sample Collection

The trial was carried out using a total of 270 juvenile European sea bass (*Dicentrarchus labrax*) reared in a commercial farm (Gargano Pesca, Manfredonia, Apulia, South Italy; 41°37′14′′ N, 15°56′57′′ E). After an adaptation period of two weeks, the fish were introduced in three recirculating seawater systems equipped with 3 fiberglass cylindrical tanks of 250 L water capacity each (for a total of 9 tanks), supplied with a continuous flow of filtered seawater. During the trial, water temperature was monitored daily (24.0 ± 0.5 °C) and salinity averaged 35 ± 1‰. After 2 weeks of adaptation to the experimental conditions, homogeneous groups of 30 fish each with an initial mean body weight of 15.0 g (± 0.2) were randomly distributed to each tank. Diets were randomly assigned to triplicate groups of these fish. During the trial, fish were fed by hand, two times a day (at 10:00 and 16:00 o’clock) to apparent visual satiation. Fish were treated according to the “Council Directive 86/609 EEC for the protection of animals used for experimental and other scientific purposes” and to the “Ethical Justification for the Use and Treatment of Fishes in Research” [33].

The growth trial lasted three months; at the end of the experiment, fish were caught by net and slaughtered by immersing in ice-cold water (hypothermia), accordingly to the laws in force [34]. The fish were immediately placed on ice and transported to the laboratory in refrigerated conditions. The following parameters were calculated:

- Survival rate (%) = [number of fish stocked − number of dead fish harvested/number of fish stocked] × 100;
- Weight gain (g/fish) = [final mean body weight (W2) − initial mean body weight (W1)]/days of trial (T);
- Specific growth rate (SGR, %) = 100 × (ln W2 − ln W1)/T [35].

### 2.3. pH, Colour, and Textural Parameters in Sea Bass Fillets

The pH values were measured on the fish fillets using a portable instrument (Model HI 9025; Hanna Instruments, Woonsocket, RI, USA) with an electrode (FC 230C; Hanna Instruments) and performing a two-point calibration (pH 7.01 and 4.01). The colorimetric features (L * = lightness, a * = redness, b * = yellowness) of the fish fillets were determined using a Hunter Lab Miniscan™ XE Spectrophotometer (Model 4500/L, 45/0 LAV, 3.20 cm diameter aperture, 10° standard observer, focusing at 25 mm, illuminant D65/10; Hunter Associates Laboratory Inc., Reston, VA, USA) by taking three readings for each sample along the whole fillet of the left side (in correspondence of the cranial, middle, and caudal fin regions). The instrument was normalized to a standard white tile provided with the instrument before performing analysis (Y = 92.8, x = 0.3162 and y = 0.3322) [36].

Rheological properties of the raw fish fillets were assessed using an Instron 5544 Universal Testing Machine (Instron Corp., Canton, MA, USA). Texture Profile Analysis (TPA) was performed using a flat steel probe of 25 mm diameter, through a double compression test elaborated by the incorporated software. From the left side of each fish, three samples with a square surface (2 × 2 cm) and a height of 0.5 cm were excised in three different locations along the whole fillet (as for colour assessment). Mean values of measurements of each test per animal were retained for statistical analysis. The fillet samples underwent two compression cycles, 2 minutes apart, at a speed rate of 0.6 m/s. A compression equal to 6% of the initial height of the sample was exerted for both cycles. The following parameters were recorded: resistance at maximum compression during the first cycle (N), which is the force necessary to attain a given deformation and represents the hardness of the sample at the first bite; resistance at maximum compression during the second cycle (N), which represents the hardness of the sample at the second bite; springiness, i.e., elasticity, which is the rate at which a deformed sample returns to its original size and shape (mm); cohesion force resilience (no unit, F2/F1), i.e., strength of the internal bonds in the sample; and chewiness, namely the energy required to chew a solid sample to a steady state of swallowing (N * mm) [37].

### 2.4. Chemical and Fatty Acid Analysis of Fillets

Fillets held in ice were rapidly skinned, chopped, combined in a pool, and homogenized for 1 min. AOAC procedures were used to assess the moisture, ether extract, raw protein, and ash [38]. The total lipids were extracted using a 2:1 chloroform/methanol (*v/v*) solution to determine the fatty acid profile [39]. The fatty acids were then methylated using a KOH/methanol 2N solution [40] and analysed by gas chromatography (Shimadzu GC-17A) using a silicone-glass capillary column (70% Cyanopropyl Polysilphenylene-siloxane BPX 70 by Thermo Scientific, length = 60 m, internal diameter = 0.25 mm, film thickness = 0.25 µm). The starting temperature was 135 °C for 7 min, then the temperature was increased by 4 °C/min up to 210 °C. Fatty acids were identified by comparison of retention times to authentic standards for percentage area normalization. Fatty acids were expressed as percentage (wt/wt) of total methylated fatty acids.

The food risk factors of meat were determined by calculating the Atherogenic (AI) and Thrombogenic (TI) Indices [41]:

AI = [(C12:0 + 4 x C14:0 + C16:0)] − [ΣMUFA + Σn−6 + Σn−3];

TI = [(C14:0 + C16:0 + C18:0)] − [(0.5 × ΣMUFA + 0.5 × Σn−6 + 3 × Σn−3 + Σn−3)/Σn−6];

where MUFA are monounsaturated fatty acids.

Lipid oxidation was evaluated on fillet samples stored at 4 °C for 48 h after slaughtering by measuring the concentration of 2-thiobarbituric acid reactive substances (T-BARS) [42] and expressed as mg malondialdehyde (MDA)/kg meat. The test is based on a spectrophotometric quantification (532 nm) of the reaction occurring between MDA and thiobarbituric acid (TBA), in conditions of high temperature and low pH, which determine the formation of a pink coloured complex.

### 2.5. Statistical Analysis

The data were subjected to statistical analysis of variance (ANOVA) using a one-way test to evaluate the dietary treatments [43]. Differences among diets were compared using Duncan’s test, and significance was declared at (*p* < 0.05). Data are reported as means and standard error of means (SEM). 

## 3. Results and Discussion

### 3.1. Growth Parameters

No differences arose between dietary treatments for the fish survival rate, which was on average equal to 94.7% ± 0.3. The final body weight was recorded, and the specific growth rate for fish of the GPE 200 group was significantly lower (*p* < 0.05) in comparison to the control group (Table 2). The final body weights recorded in this study were comparable to those reported by Moreira et al. [44] for juvenile sea bass farmed at 25 °C for a period of less than two weeks compared to our trial. The specific growth rates of our experiment are similar to those observed by other authors [35,44].

### 3.2. pH, Colour and Textural Parameters in Sea Bass Fillets

The diet did not affect the L value of fish fillets, which was similar among groups (Table 3). Dietary inclusion of the red grape extract, at both the tested concentrations, determined a significant increase (*p* < 0.05) of the red index (a *) and lower yellow values (b *) as compared to the control group. The different redness value of fish fillets from the GPE 100 and GPE 200 groups may be due to the dietary effect of red grape extracts, which are naturally rich in colourants, as found in other studies that reported meat colour intensification following diets containing red grape extracts [45,46]. Turcu et al. [46] found that supplementation with grape pomace to broilers fed PUFA-enriched diets determined lower lightness and higher redness and yellowness values, although in a different way for breast and thigh meat, since the former is mainly composed of white muscle fibres (low in myoglobin), while the latter is made of red fibres (richer in myoglobin). Some authors [47] observed that red grape antioxidant dietary fibre added to minced fish muscle raised the value of a * but not that of b *. Due to their strong antioxidant activity, phenols may influence meat colour, and changes in the redness values have been related to the antioxidant effect exerted on blood myoglobin while variations in the yellow index is a result of decreased lipid oxidation, as thoroughly reviewed by Mainente et al. [48].

Dietary inclusion of red grape extracts significantly (*p* < 0.05) lowered fillet hardness, regardless of the concentration used, in comparison with the control diet, during both compression cycles (Table 2). While springiness and cohesion force resilience were unaffected by the diet, the chewiness of control fish fillets was significantly greater (*p* < 0.05) in comparison to both the GPE groups. Similarly, some authors [47] found that the addition of grape pomace powders to minced fish muscle determined a significant decrease of the shear force and chewiness. On the other hand, other researchers [49] found similar shear forces in cooked pork sausages containing 1% of grape pomace powders up to 3 days of storage, while prolongation of storage showed higher shear force values in grape pomace-added samples, probably due to the slow lipid oxidation promoted by pro-oxidant flavonoids present in grape extracts.

The MDA content results from the oxidation of carbon–carbon double bond of polyunsaturated fatty acids [50]. Free radicals are known to induce lipid peroxidation, playing an important role in pathological processes in vita as well as on the shelf-life of animal products. Therefore, MDA has been pointed out as the main product to evaluate lipid peroxidation. The TBARS test is the most used method for the identification of occurrence of lipid degradation that measures the production of secondary lipid oxidation compounds, represented by the formation of MDA. In this experiment, the MDA content of fish fillets was significantly lower (*p* < 0.05) following the dietary inclusion of red grape polyphenol extracts as compared to the control group (Table 2). The antioxidant effect was not different for the two levels of GPE dietary supplementation, thus showing that the protective effect of the extract against oxidation is widely guaranteed by the lower concentration. Other studies reported that samples without grape extracts showed higher concentration of oxidation products as a result of peroxide decomposition, formation of carbonyls, and the interaction of these compounds with nucleophilic molecules, as free amino acids, peptides, proteins, and aminated phospholipids present in the muscle [47].

### 3.3. Chemical Composition and Fatty Acid Profile of Fillets

The chemical composition of fish fillets is shown in Table 4. In the present study, the diet affected only the total lipid content of fish, which was significantly lower (*p* < 0.05) in both the groups fed with the red grape extract as compared to the control one. The effects of polyphenol-enriched diets in fish species on the chemical composition of fillets seems quite controversial. Kesbiç et al. [51] reported that the nutrient contents of rainbow trout fed grape seed extracts at various rates had no significant effect on fillet fat or moisture and ash contents, whereas dietary inclusion of 0.5‰ grape seed extract determined a significant increase in the fillet–protein ratio. Other authors [52] reported that supplementation with GP meal in rainbow trout feed led to decreased total lipid and ash, while protein content remained relatively unchanged regardless of the supplementation level. Some researchers [53] found that dietary inclusion of polyphenolic tannins markedly lowered the lipid but not the protein content of seabass fillets, accordingly to our findings. The authors hypothesized that the depletion of lipids following polyphenol-enriched diets may be due to the inhibition of lipid synthesis and/or to enhanced lipid mobilization.

The fatty acid profile of sea bass fillets is shown in Table 5. Feed is one of the most important factors affecting the muscle tissue and lipid content of fish [54]. In this study, the total concentration of SFAs was significantly higher (*p* < 0.05) in the control as compared to the GPE groups in sea bass and consistent with that reported by other authors [55,56].

Adversely, other researchers [54] found that the SFA concentration of fillets proportionally increased with the grape seed oil content in the fish diet. Different authors [57,58] described that the dietary supplementation with grape seed oil reduced SFA while enriching chicken meat with n-3 fatty acids.

On the other hand, though lipids in grapes are primarily present in seeds, out of which 90% accounts for MUFAs [59,60], in our study no differences among groups were found for the MUFA content in fish fillets.

As for the long chain PUFAs, the concentration of arachidonic acid (C20:4 n-6) was significantly (*p* < 0.05) higher in both the GPE groups and similar to the results reported in a previous study carried out on wild sea bass [61]. Likewise, the content of EPA (C20: n-3), DPA (C22:5 n-6), and DHA (C22:6 n-3) was also markedly higher in fillets from fish fed with the grape seed extract. These fatty acids have an important nutritional role since they exert beneficial effects on the brain and retina development during the early stage of human life [61].

The total PUFA concentration was significantly (*p* < 0.05) higher in the GPE groups as compared to the control. However, no differences were found neither for the n-6/n-3 nor for the n-3/n-6 ratio, whose values fell within the ranges reported in other studies [56,62]. On the contrary, other authors [54] reported that the n-3/n-6 ratios significantly decreased by increasing supplementation of grape seed oil in experimental diets. Some researchers reported that the change in fatty acid ratios in fish meat depends on metabolic factors, such as digestibility and fatty acid catabolism [63]. 

The Atherogenic index (AI) and Thrombogenic index (TI) of sea bass fed with the experimental diets are given in Table 4. These two indices express the nutritional quality of foods and the potential effects of fats on the occurrence and development of coronary diseases [41]. Low levels of myristic, palmitic, and stearic acids in foods are known to be beneficial to human health. Similarly, low AI and TI values in food provide protection against cardiovascular diseases [63,64]. In the present study, AI values in fish flesh significantly (*p* < 0.05) decreased in the groups fed diets containing GPE, while no differences among groups were found for the TI. The AI values found in this experiment were similar to those obtained in wild sea bass as reported by Tarricone et al. [56].

## 4. Conclusions

This a preliminary study carried out on juvenile sea bass in order to ascertain whether dietary inclusion of a red grape polyphenol-rich extract affects the quality of fillets. As for the level of inclusion of the GPE extract, the lower concentration used (100 mg/kg) provided better results as compared to the higher level (200 mg/kg).

The overall texture of fish fillets, which is an important feature for consumers, was improved following dietary GPE. Furthermore, dietary red grape extract lowered the total lipid content of fish, the concentration of saturated fatty acids, and the Atherogenic index while it determined an increase of the content of polyunsaturated fatty acids, with great benefits for human health. Fillets from both the GPE groups showed a lower concentration of MDA, thus confirming the antioxidant effect of the polyphenol-rich extract and its effectiveness in preventing lipid oxidation.

Further investigation is needed in order to study the effects of dietary inclusion of the red grape extract (100 mg/kg) on fish performances, immune status, and eating quality upon achievement of the sea bass market size.

## Figures and Tables

**Table 1:** Ingredients, chemical composition, and fatty acid profile of the conventional feed.

Item	g/kg
Fish meal (65%)	381.3
Corn gluten	259.8
Wheat meal	150.7
Lysin (99%)	7.2
Fish oil	176
Premix ^1^	25.0
**Proximate composition (% on DM basis)**	
Moisture	10.0
Crude protein	48.0
Total lipid	21.5
Ash	8.0
Crude fibre	1.5
**Fatty acid profile (% FA methyl esters)**	
C14:0 (myristic)	5.1
C15:0 (pentadecanoic)	0.4
C16:0 (palmitic)	15.8
C18:0 (stearic)	5.1
C16:1 n7 (palmitoleic)	5.4
C18:1 n9 (oleic)	16.5
C20:1 n9 (eicosanoic)	2.9
C18:2 n6 (linoleic)	11.3
C18:3n6 (γ-linolenic)	1.1
C18:3n3 (α-linolenic)	1.9
C18:4n3 (stearidonic)	1.6
C20:4n6 (arachidonic, ARA)	0.7
C20:5n3 (eicosapentaenoic, EPA)	7.9
C22:5n6 (docosapentaenoic, DPA)	0.3
C22:5n3 (docosapentaenoate)	0.4
C22:6n3 (docosahexaenoic, DHA)	10.2

^1^ Premix provides per kg: Vitamin A (400,000 IU), Vitamin D_3_ (100,000 IU), Vitamin E (230 mg), Vitamin K_3_ (165 mg), Vitamin B_1_ (300 mg), Vitamin B_2_ (80 mg), Vitamin B_3_ (1000 mg), Vitamin B_6_ (200 mg), Vitamin B_9_ (100 mg), Vitamin B_12_ (1 mg), Biotin (2 mg), Pantothenic acid (220 mg), Vitamin C (650 mg), Cupric sulphate (900 mg), Iron sulphate (330 mg), Magnesium sulphate (1000 mg), Cobalt sulphate (100 mg), Choline chloride (10,000 mg), Potassium iodide (50 mg), Manganese oxide (960 mg), Sodium selenite (1 mg), Zinc sulphate (750 mg), Calcium carbonate (1000 mg).

**Table 2:** Growth performances of European sea bass fed diets with two levels of red grape extracts.

Parameters	Control	GPE 100	GPE 200	SEM ^1^	*p*-Value
Initial body weight (W1, g)	15.02	15.01	15.03	0.01	-
Final body weight (W2, g)	50.14 ^a^	48.92 ^ab^	46.14 ^b^	2.25	0.045
Weight gain (g/d)	0.42	0.40	0.37	0.03	0.062
Specific growth rate (%)	1.44 ^a^	1.41 ^ab^	1.34 ^b^	0.41	0.047

GPE 100: red grape polyphenol extract 100 mg/kg; GPE 200: red grape polyphenol extract 200 mg/kg. ^1^ Standard error of means; Differences between diets: ^a, b^: *p* < 0.05.

**Table 3:** Colour features, texture profile analysis, and MDA concentration in fillets from European sea bass fed diets with two levels of red grape extracts.

Parameters	Control	GPE 100	GPE 200	SEM ^1^	*p*-Value
L * (lightness)	32.07	31.99	32.62	0.28	0.201
a * (redness)	−2.59 ^b^	−2.21 ^a^	−2.35 ^a^	0.19	0.035
b * (yellowness)	3.30 ^a^	2.85 ^b^	2.53 ^b^	0.36	0.035
pH	6.35	6.31	6.32	0.02	0.108
Maximum compression 1st cycle (F1)	104.47 ^a^	77.96 ^b^	76.30 ^b^	23.07	0.028
Maximum compression 2nd cycle (F2)	78.73 ^a^	54.83 ^b^	55.51 ^b^	17.93	0.031
Springiness (mm)	1.86	1.65	1.70	0.13	0.103
Cohesion force resilience (F2/F1)	0.74	0.73	0.73	0.01	0.125
Chewiness (N*mm)	14.82 ^a^	7.38 ^b^	7.91 ^b^	3.39	0.020
Malondialdehyde (MDA, mg/kg)	0.044 ^a^	0.032 ^b^	0.032 ^b^	0.01	0.029

GPE 100: red grape polyphenol extract 100 mg/kg; GPE 200: red grape polyphenol extract 200 mg/kg. ^1^ Standard error of means. Differences between diets: ^a, b^: *p* < 0.05.

**Table 4:** Chemical composition of fillets from European sea bass fed diets with two levels of red grape extracts.

Parameters	Control	GPE 100	GPE 200	SEM ^1^	*p*-Value
Moisture	72.27	73.33	73.04	1.57	0.092
Crude protein	18.54	19.08	18.98	0.72	0.086
Lipid	6.33 ^a^	4.71 ^b^	4.79 ^b^	1.49	0.043
Ash	1.62	1.46	1.93	0.30	0.061
N Free-Extract	1.24	1.42	1.26	0.48	0.062

GPE 100: red grape polyphenol extract 100 mg/kg; GPE 200: red grape polyphenol extract 200 mg/kg. ^1^ Standard error of means. Differences between diets: ^a, b^: *p* < 0.05.

**Table 5:** Fatty acid profile of fillets from European sea bass fed diets with two levels of red grape extracts (% total fatty acids methyl esters).

Fatty Acids	Control	GPE 100	GPE 200	SEM ^1^	*p*-Value
C12:0 (lauric)	0.07 ^a^	0.05 ^b^	0.04 ^b^	0.01	0.043
C14:0 (myristic)	5.94 ^a^	4.60 ^b^	4.54 ^b^	0.12	0.045
C15:0 (pentadecylic)	0.69 ^a^	0.48 ^b^	0.48 ^b^	0.02	0.048
C16:0 (palmitic)	22.04 ^a^	20.91 ^b^	20.86 ^b^	0.54	0.039
C17:0 (heptadecanoic)	0.59	0.81	0.80	0.03	0.131
C18:0 (stearic)	4.39 ^a^	3.21 ^b^	3.18 ^b^	0.11	0.035
Total SFA ^2^	33.72 ^a^	30.06 ^b^	29.90 ^b^	0.62	0.029
C16:1 n-7 (palmitoleic)	7.19	7.56	7.48	0.15	0.089
C16:1 n-9 (trans9-palmitoleic acid)	0.79 ^b^	1.12 ^a^	1.14 ^a^	0.04	0.039
C17:1 (ginkgolic)	0.38 ^b^	0.66 ^a^	0.71 ^a^	0.03	0.031
C18:1 n-7 (cis-vaccenic acid)	3.09 ^b^	5.04 ^a^	5.30 ^a^	0.08	0.027
C18:1 n-9 (oleic)	20.01	19.45	19.30	0.35	0.094
C20:1 n-9 (eiocosanoic)	4.81 ^a^	2.38 ^b^	2.54 ^b^	0.07	0.021
Total MUFA ^3^	36.27	36.21	36.47	0.54	0.100
C18:2 n-6 (linoleic)	6.12	5.82	5.64	0.19	0.098
C18:3 n-3 (α-linolenic)	0.42 ^b^	0.67 ^a^	0.70 ^a^	0.03	0.043
C18:3 n-6 (γ-linolenic)	0.49 ^b^	0.74 ^a^	0.74 ^a^	0.03	0.038
C20:4 n-3 (eicosatetraenoico)	0.52	0.63	0.64	0.06	0.103
C20:4 n-6 (arachidonic)	3.74 ^b^	4.32 ^a^	4.25 ^a^	0.12	0.040
C20:5 n-3 (eicosapentaenoic, EPA)	5.56 ^b^	6.21 ^a^	6.19 ^a^	0.28	0.041
C22:5 n-6 (docosapentaenoic, DPA)	0.35 ^c^	0.68 ^b^	0.79 ^a^	0.08	0.029
C22:5 n-3 (docosapentaenoate)	0.99 ^b^	1.54 ^a^	1.60 ^a^	0.07	0.025
C22:6 n-3 (docosahexaenoic, DHA)	11.82 ^b^	13.12 ^a^	13.08 ^a^	0.72	0.038
Total PUFA ^4^	30.01 ^b^	33.73 ^a^	33.63 ^a^	0.95	0.035
Total n-6 ^5^	10.70 ^b^	11.56 ^a^	11.42 ^a^	0.98	0.045
Total n-3 ^6^	19.31 ^b^	22.17 ^a^	22.21 ^a^	0.23	0.035
n-3/n-6	1.80	1.92	1.94	0.10	0.149
n-6/n-3	0.55	0.52	0.51	0.09	0.105
AI (Atherogenic index)	0.69 ^a^	0.56 ^b^	0.55 ^b^	0.02	0.042
TI (Thrombogenic index)	0.39	0.31	0.30	0.01	0.108

GPE 100: red grape polyphenol extract 100 mg/kg; GPE 200: red grape polyphenol extract 200 mg/kg. ^1^ Standard error of means. Differences between diets: ^a, b^*: p* < 0.05. ^2^ Total SFA—saturated fatty acids (sum of C12:0 + C14:0 + C15:0 + C16:0 + C17:0 + C18:0); ^3^ Total MUFA—monounsaturated fatty acids (sum of C16:1 n7 + C16:1 n9 + C17:1 + C18:1 n7 + C18:1 n9+ C20:1 n9); ^4^ Total PUFA—polyunsaturated fatty acids (sum of n-6 + n-3); ^5^ Total n-6 (sum of C18:2 n6 + C18:3 n6 + C20:4 n6 + C22:5 n6); ^6^ Total n-3 (sum of C18:3 n3+ C20:4 n3 + C20:5 n3 + C22:5 n3 + C22:6 n3); ^a, b, c^: *p* < 0.05.

## Data Availability

The datasets used and/or analysed during the current study are available from the first author and from the corresponding author on reasonable request.
